# Regulation of CD137 expression through K-Ras signaling in pancreatic cancer cells

**DOI:** 10.1186/s40880-019-0386-4

**Published:** 2019-07-09

**Authors:** Christophe Glorieux, Peng Huang

**Affiliations:** Sun Yat-sen University Cancer Center, State Key Laboratory of Oncology in South China, Collaborative Innovation Center for Cancer Medicine, Guangzhou, 510060 Guangdong P. R. China

**Keywords:** CD137, K-Ras, IL-1α (interleukin-1 alpha), MAPK (mitogen-activated protein kinases), NF-κB (nuclear factor kappa-light-chain-enhancer of activated B cells), Pancreatic cancer, Oxidative stress, Soluble CD137

## Abstract

**Background:**

The interaction between CD137 and its ligand (CD137L) plays a major role in the regulation of immune functions and affects cancer immunotherapy. CD137 is a cell surface protein mainly located on activated T cells, and its regulation and functions in immune cells are well established. However, the expression of CD137 and its regulation in cancer cells remain poorly understood. The main purposes of this study were to examine the expression of CD137 in pancreatic cancer cells and to investigate its underlying mechanisms.

**Methods:**

Cells containing inducible K-Ras^G12V^ expression vector or with different K-Ras mutational statuses were used as in vitro models to examine the regulation of CD137 expression by K-Ras. Various molecular assays were employed to explore the regulatory mechanisms. Tumor specimens from 15 pancreatic cancer patients and serum samples from 10 patients and 10 healthy donors were used to test if the expression of CD137 could be validated in clinical samples.

**Results:**

We found that the CD137 protein was expressed on the cell surface in pancreatic cancer tissues and cancer cell lines. Enzyme-linked immunosorbent assay revealed no difference in the levels of secreted CD137 in the sera of patients and healthy donors. By using the K-Ras inducible cell system, we further showed that oncogenic K-Ras up-regulated CD137 through the activation of MAPK (mitogen-activated protein kinases) and NF-κB (nuclear factor kappa-light-chain-enhancer of activated B cells) pathways, as evidenced by significantly reduced CD137 mRNA expression led by genetic silencing of MAPK1 and p65, the key proteins involved in the respective pathways. Furthermore, we also found that the NF-κB pathway was mainly stimulated by the K-Ras-induced secretion of interleukin-1α (IL-1α) which promoted the transcription of the *CD137* gene in pancreatic cancer cell lines. Analysis of the TCGA (the cancer genome atlas) database also revealed a significant correlation between IL-1α and CD137 expression (*r* = 0.274) in tumor samples from pancreatic cancer patients (*P* < 0.001).

**Conclusions:**

The present study has demonstrated that the CD137 protein was expressed on pancreatic cancer cell surface, and has identified a novel mechanism by which K-Ras regulates CD137 in pancreatic cancer cells through MAPK and NF-κB pathways stimulated by IL-1α.

## Background

Activation of K-Ras by mutation is frequently observed in human tumors, especially in pancreatic cancer where approximately 90% of malignant cells exhibit constitutive activation of K-Ras [1, 2]. In lung [3, 4] and colon cancers [5], K-Ras mutations are also frequent (30%–40%). The presence of K-Ras mutations in cancer cells is associated with disease progression and poor outcome in part due to the activation of several downstream pathways that induce cell proliferation, tumor cell survival, and invasion [6, 7].

Evasion of immune surveillance is another important feature of cancer, which express certain molecules that affect immune functions. Secretion of cytokines and expression of PD-L1 (programmed death-ligand 1) by cancer cells are well-known to modulate the immune system and play important roles in cancer development. The tumor necrosis factor receptor superfamily member 9 (TNFRSF9), also known as CD137 or 4-1BB, is another important immune-modulating molecule known to be expressed on the surface of certain immune cells. CD137 was originally discovered in 1989 and reported as a cell surface protein mainly located on activated CD4^+^ and CD8^+^ T cells [8, 9]. Its expression is regulated by certain transcription factors [the activator protein 1 (AP-1) and nuclear factor kappa-light-chain-enhancer of activated B cells (NF-κB) as well as cytokines [interleukin-2 (IL-2) and IL-4] in activated T cells [10–12] and possibly in other immune cells [13]. Interaction of CD137 with its ligand (CD137L or 4-1BBL) on activated antigen-presenting cells could lead to bidirectional activation that promotes immunity against cancer [14, 15]. For instance, it has been shown that signals through the CD137 receptor are delivered by the agonistic CD137 antibodies and CD137L overexpression, and these signals could lead to T cell activation and survival [15]. In the reverse direction, engaged CD137L could impact its expressing cells such as dendritic cells, leading to their activation, maturation, and differentiation [16]. Hence, the use of agonistic CD137 antibodies (i.e., urelumab and utomilumab) is considered as a promising immunotherapeutic approach to treat various types of tumors [17].

Although the expression of CD137 in lung cancer [18], leukemia [19], and lymphoma [20] cells have been reported, the molecular mechanisms that regulate *CD137* gene expression in cancer cells are still poorly understood and remain to be elucidated. In this study, we aimed to test if CD137 is expressed in human pancreatic cancer cells and to search for its regulatory mechanisms.

## Materials and methods

### Cell lines and cell culture

The doxycycline-inducible T-Rex/K-Ras^G12V^ cells (source: fetus) were constructed as previously described [21] and cultured in Dulbecco’s modified Eagle’s medium (DMEM) supplemented with 10% tetracycline-free fetal bovine serum (FBS). Panc-1 (source: male), SW1990 (source: male), HCT116 (source: male), and the hTERT (human telomerase reverse transcriptase) immortalized HPNE cell lines (source: male) were purchased from the American Type Culture Collection (ATCC, Manassas, VA, USA). They were cultured in DMEM with 10% FBS, except for HCT116 which was cultured in McCoy’s 5A medium with 10% FBS. HPNE cells stably transfected with mutant K-Ras^G12V^ were provided by Prof. Paul Chiao (MD Anderson Cancer Center, Houston, TX, USA) and cultured in DMEM with 10% FBS as previously described [22]. All cell lines were confirmed to be mycoplasma-negative (LookOut mycoplasma polymerase chain reaction [PCR] detection kit, Sigma, St. Louis, MO, USA), and authentication of cell lines was performed by STR (short tandem repeats) genotyping (Microread Genetics, Beijing, China). Doxycycline, glucose oxidase, and catalase were purchased from Sigma (St. Louis, MO, USA). Human recombinant IL-1α was from Thermo Fisher Scientific (Rockford, IL, USA). Neutralizing IL-1α antibody was from R&D systems (Minneapolis, MN, USA; #MAB200).

### Human samples

Human pancreatic cancer tissues were provided by Shanghai Outdo Biotech (Shanghai, China) as a sample array containing 15 de-identified tumor samples. De-identified serum samples from healthy donors and pancreatic cancer patients were obtained from the tissue bank of Sun Yat-sen University Cancer Center (Guangzhou, Guangdong, China). Studies using de-identified human samples were reviewed and approved by Committee for Ethical Review of Research involving Human Subjects of Sun Yat-sen University.

### Quantitative reverse transcription-polymerase chain reaction

Total RNA was isolated using Trizol (Invitrogen, Grand Island, NY, USA) according to the manufacturer’s instructions. RNA was reversely transcribed using the Primer Script RT reagent Kit with gDNA Eraser (Takara Bio Inc, Kusatsu, Shiga, Japan). The primer sequences for human CD137 were 5′-TCCGCAGATCATCTCCTTCT-3′ (forward) and 5′-AGTTTCTTTCTGCCCCGTTT-3′ (reverse). The primer sequences for human IL1A were 5′-TGTGACTGCCCAAGATGAAG-3′ (forward) and 5′-CCCAGAAGAAGAGGAGGTTG-3′ (reverse). The elongation factor 1 (EF1) was used as the reference gene; primer sequences for EF1 were 5′-GCTTCACTGCTCAGGTGAT-3′ (forward) and 5′-GCCGTGTGGCAATCCAAT-3′ (reverse). Real-time PCR was performed using the SYBR Premix Ex Taq RNase H+ kit (Takara) and analyzed using the Bio-Rad detection system (Bio-Rad, Hercules, CA, USA). The samples were first incubated for 5 min at 95 °C, followed by 40 cycles of 10 s at 95 °C and 30 s at 60 °C. The results were calculated using the formula 2^−(Ct target−Ct EF1)^ and matched to the control samples.

### ELISA

The level of soluble CD137 (sCD137) in serum samples from pancreatic cancer patients and healthy donors was measured using enzyme-linked immunosorbent assay (ELISA) kit (Ray Biotech, Norcross, USA; #ELH-TNFRSF9) according to the protocol provided by the manufacturer.

### Cell transfection

The small interfering RNAs (siRNAs) against MAPK1 and p65 (RelA) were designed and synthesized by RiboBio (Guangzhou, Guangdong, China). The sequences of siRNAs against MAPK1 are 5′-CGAGCAAATGAAAGATGTA-3′ and 5′-CAAGAAGACCTGAATTGTA-3′. The sequences of siRNAs against p65 are 5′-CTTCCAAGTTCCTATAGAA-3′ and 5′-GGACATATGAGACCTTCAA-3′. Cells were incubated with doxycycline to induce K-Ras expression for 48 h before siRNA transfection, using lipofectamine RNAi Max reagents (Invitrogen) according to the manufacturer’s instructions. Transfection was performed for 24 h with a 100 nmol/L siRNA solution in the presence of doxycycline. Assays for the expression of target molecules were performed 48 h after the transfection.

### Flow cytometry

For the detection of membrane CD137, cells were fixed with 4% formaldehyde in phosphate buffer saline (PBS) and stained with primary antibodies with a dilution of 1:100 for 2 h at room temperature. Rabbit anti-human CD137 antibody (#62634; Cell Signaling, Danvers, MA, USA) was used. Cells were then washed and incubated for 30 min at room temperature with PBS containing anti-rabbit immunoglobulin G (IgG) (#11-4839; eBioscience, San Diego, CA, USA) antibody coupled with FITC. Cells were then collected and washed twice with PBS before flow cytometry analysis (Gallios; Beckman Coulter, Brea, CA, USA). For each experiment, at least 10,000 cells per sample were analyzed using FlowJo software (https://www.flowjo.com).

### Immunohistochemistry

Pancreatic cancer tissue microarrays (Shanghai Outdo Biotech, Shanghai, China) were first dried at 58 °C for 1 h, dewaxed and rehydrated before epitope retrieval by heating at 100 °C in 10 mmol/L sodium-citrate (pH6.0) for 4 min. The sections were cooled down to room temperature for 30 min. To eliminate the endogenous peroxidase and alkaline phosphatase activity, the tissue sections were treated with 3% hydrogen peroxide for 20 min. The sections were then incubated with individual primary antibodies overnight, followed by incubation with secondary antibodies for 1 h. 3,3′-diaminobenzidine (DAB) was then applied as a substrate to reveal the antigen. Hematoxylin was used for counterstaining. The primary antibody used in this study was rabbit anti-CD137 (#62634; Cell Signaling). All other reagents were from ZSGB-Bio (Beijing, China).

### Bioinformatics

Illumina HiSeq_RNASeqV2 RSEM normalized gene expression profiles for human pancreatic adenocarcinoma were retrieved from The Cancer Genome Atlas (TCGA) Pan-Cancer atlas (paad_tcga_pan_can_atlas_2018) using the Cancer Genomics Data Server (CGDS)-R (R functions for querying the CGDS) package. A total of 166 samples with expression data for CD137 and IL1A were included for analysis.

### Statistical analysis

All experiments were repeated at least three times. Q–Q plots were used to compare and determine data distribution. Data are expressed as mean ± SEM (standard error of the mean) unless otherwise specified. Student *t*-tests were used to evaluate the statistical significance of the difference between two groups of samples with normal distributions. Despite a large sample size, the relationship between CD137 and IL1A expression in human pancreatic carcinoma tissues was assessed using a Spearman’s rank correlation because of the nature of data (integer scores). Statistical analyses were performed using the GraphPad Prism software (San Diego, CA, USA). No statistical method was used to calculate sample sizes, which were determined empirically. All tests were two-tailed, and a *P* value of 0.05 or less was considered statistically significant.

## Results

### Induction of CD137 expression by oncogenic K-Ras in cancer cells

**Fig. 1:**
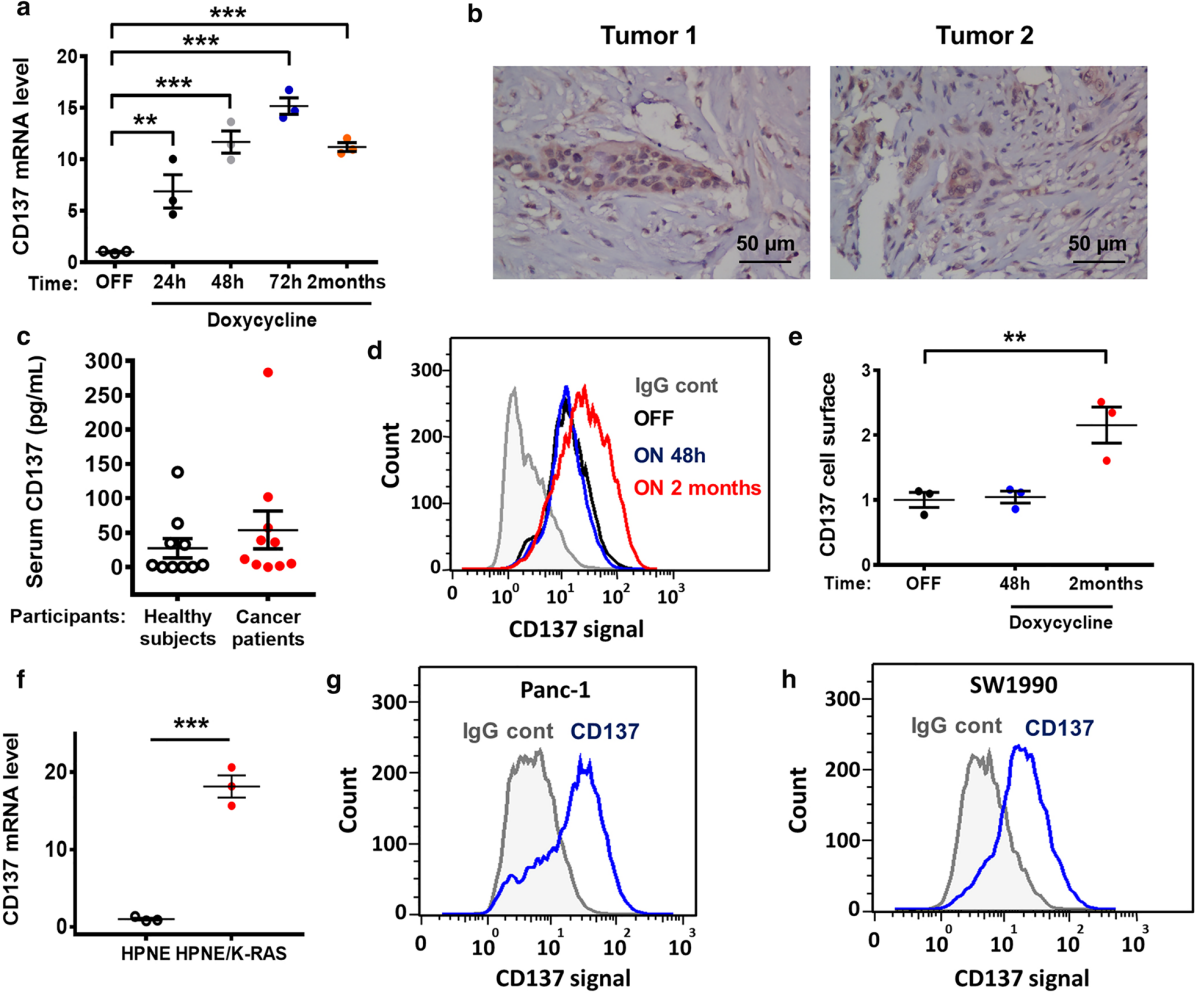
Activation of oncogenic K-Ras induces CD137 expression in cancer cells. **a** T-Rex/K-Ras cells were incubated with 100 ng/mL doxycycline for the indicated time to induce K-Ras^G12V^ expression. CD137 mRNA was measured by real-time PCR. **b** Immunostaining of CD137 in human pancreatic cancer cells (tissue microarray). **c** The levels of soluble CD137 in the sera of 10 healthy subjects and 10 pancreatic cancer patients were quantified by ELISA (enzyme-linked immunosorbent assay). **d**, **e** T-Rex/K-Ras cells incubated without (OFF) or with (ON) doxycycline for 48 h or 2 months were stained with control antibody (IgG cont) or specific antibody against human CD137. Cell surface CD137 was analyzed by flow cytometry (representative of three separate experiments). **f** Expression of CD137 in HPNE cells stably transfected with K-Ras (HPNE/K-Ras) in comparison with parental HPNE cells, measured by real-time PCR. **g**, **h** Cell surface CD137 in Panc-1 and SW1990 cell lines was analyzed by flow cytometry (representative of three separate experiments). Data are presented as mean ± SEM (standard error of the mean) of three repeated experiments. Statistical analysis: two-tailed unpaired *t*-test for panels **a**, **c**, **e** and **f**. **P < 0.01; ***P < 0.001. *CD137* tumor necrosis factor receptor superfamily member 9, *PCR* polymerase chain reaction

To explore the potential molecular pathways by which oncogenic K-Ras might affect tumor immunity, we first used cells with doxycycline-induced K-Ras^G12V^ expression, designated as T-Rex/K-Ras cells, to examine changes in gene expression profile after K-Ras activation. Surprisingly, microarray analyses revealed a 4.9-fold increase in CD137 mRNA expression 72 h after K-Ras was induced by doxycycline, which was further confirmed by real-time PCR analysis (Fig. 1a). The activation of oncogenic K-Ras^G12V^ could induce CD137 expression in a time-dependent manner and reached approximately 15-fold increase 72 h after the induction of K-Ras^G12V^. This increase in CD137 expression remained high (approximately tenfold) during a 2-month K-Ras induction (Fig. 1a). Consistently, we detected CD137 in the K-Ras-driven pancreatic cancer specimens (Fig. 1b). Since CD137 has been shown to be secreted in the serum of colon cancer patients as an immune suppressor molecule [23], we thus explored the possibility that the protein may be present in the sera of pancreatic cancer patients. Unlike colon cancer, soluble CD137 levels were barely detected in sera of pancreatic cancer patients at the levels similar to those detected in the sera of healthy individuals (Fig. 1c). Although the increase in CD137 mRNA could be detected after a 48-h K-Ras induction, the increase of CD137 protein could only be detected on the cell surface after a 2-month incubation with doxycycline (Fig. 1d, e). Similar results were also observed in hTERT-immortalized human pancreatic cancer cells (HPNE), where the expression of K-Ras^G12D^ enhanced the expression of CD137 (Fig. 1f). Moreover, the CD137 protein was detected on the surface of Panc-1 and SW1990 human pancreatic cancer cells (Fig. 1g, h).

### K-Ras-induced CD137 expression through MAPK and NF-κB pathways

**Fig. 2:**
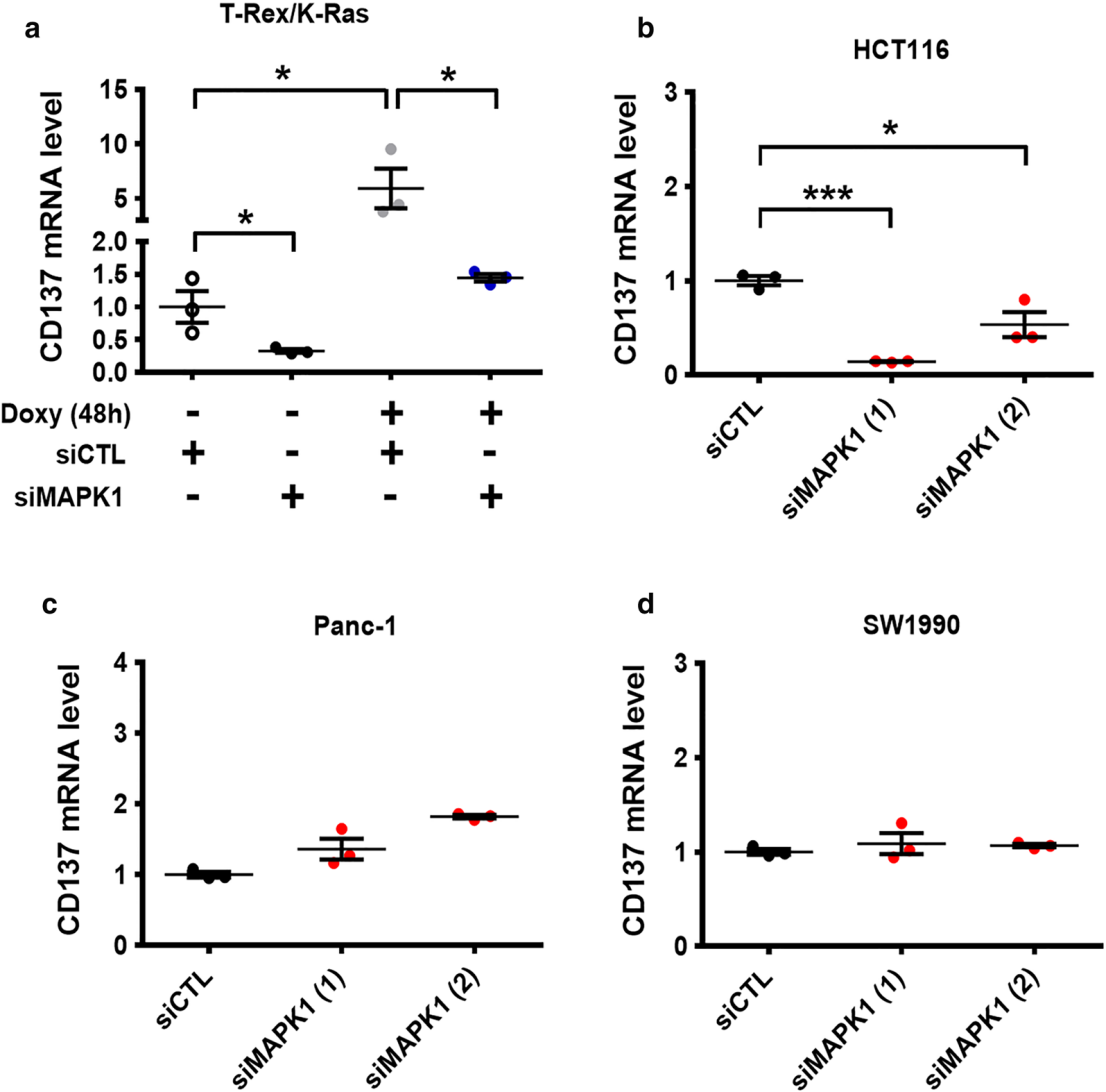
Role of MAPK signaling in mediating K-Ras-induced CD137 expression. CD137 mRNA levels in T-Rex/K-Ras (**a**), HCT116 (**b**), Panc-1 (**c**), and SW1990 cells (**d**) measured by real-time PCR. The cells were first transfected with 100 nmol/L control siRNA or siRNA against MAPK1 for 24 h, then incubated with doxycycline for 48 h. Data are presented as mean ± SEM of three repeated experiments. Statistical analysis: two-tailed unpaired *t*-test. *P < 0.05; ***P < 0.001. *CD137* tumor necrosis factor receptor superfamily member 9, *Doxy* doxycycline, *MAPK* mitogen-activated protein kinase, *siCTL* control siRNA, *siMAPK1* siRNA against MAPK1, *PCR* polymerase chain reaction

We then investigated the potential molecular mechanisms that led to CD137 up-regulation induced by oncogenic K-Ras activation. Based on our previous findings that MAPK and NF-κB signaling could be activated when K-Ras was induced by doxycycline [24], we explored the possible role of these signaling pathways in regulating the CD137 expression. As shown in Fig. 2a, the silencing of MAPK expression by siRNA significantly decreased the basal expression of CD137 in T-Rex/K-Ras-Off cells, and K-Ras activation by doxycycline suppressed the CD137 expression. MAPK1 siRNA inhibited CD137 expression consistently in human colon cancer HCT116 cell line harboring a mutated form of *KRAS* gene (Fig. 2b). Interestingly, the same MAPK1 siRNA constructs did not cause any decrease in CD137 expression in the Panc-1 (Fig. 2c) and SW1990 cells (Fig. 2d), suggesting that there could be another pathway that regulated the CD137 expression in these cells.

**Fig. 3:**
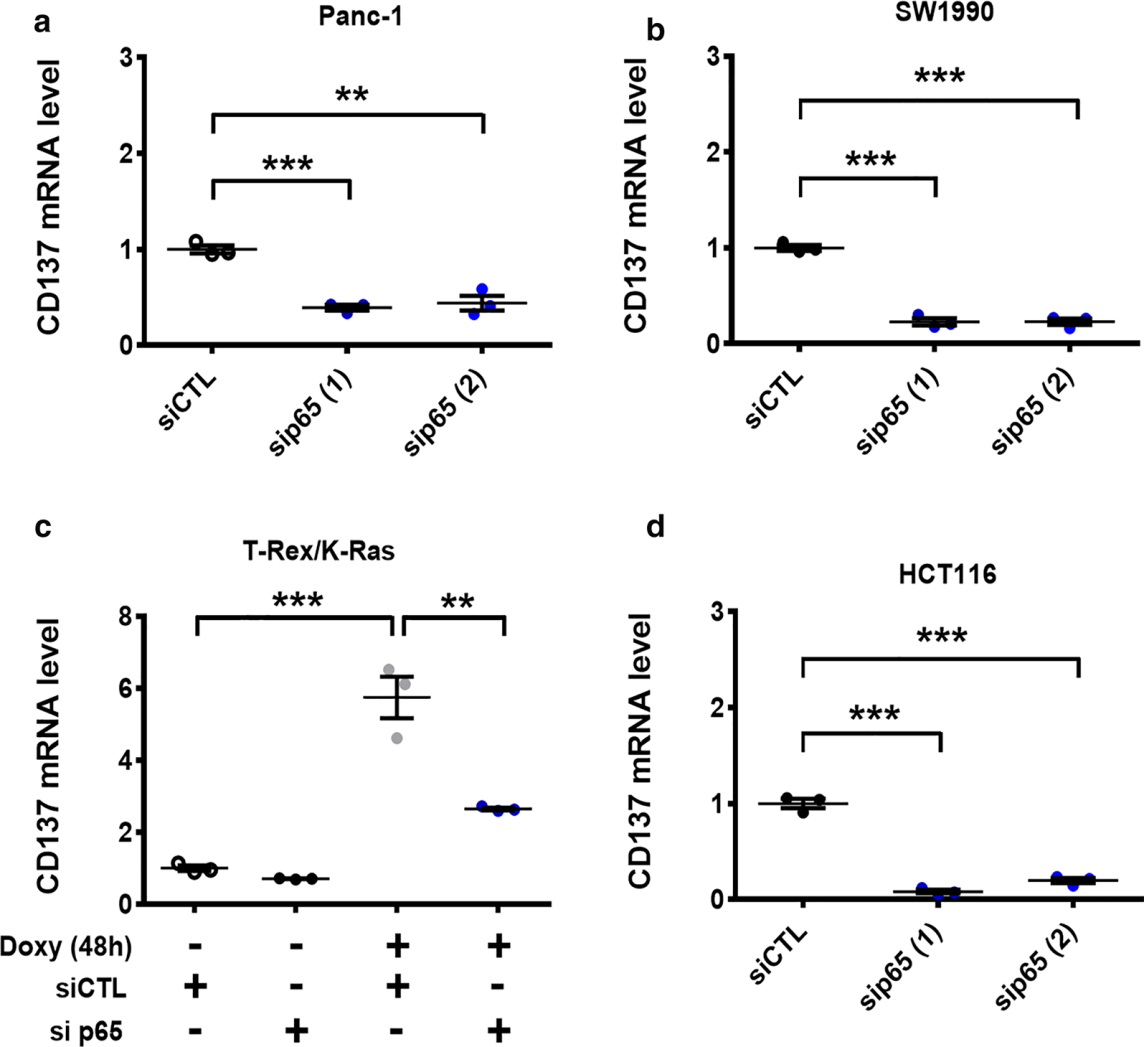
Role of the NF-κB pathway in mediating K-Ras-induced CD137 expression. **a**, **b** Panc-1 and SW1990 pancreatic cancer cells were transfected with 100 nmol/L control siRNA or siRNA against p65 (RELA) for 48 h. CD137 mRNA was measured by real-time PCR. **c** T-Rex/K-Ras cells were first transfected with 100 nmol/L control siRNA or siRNA against p65 for 24 h, then incubated with doxycycline for 48 h. CD137 mRNA was measured by real-time PCR. **d** HCT116 cells were transfected with 100 nmol/L siRNA against p65 or control RNA (siCTL) for 48 h, and CD137 mRNA was measured by real-time PCR. Data are presented as mean ± SEM of three repeated experiments. Statistical analysis: two-tailed unpaired t-test for **a**–**d**. **P < 0.01; ***P < 0.001. *CD137* tumor necrosis factor receptor superfamily member 9, *Doxy* doxycycline, *siCTL* control siRNA, *sip65* siRNA against p65, *PCR* polymerase chain reaction

We then investigated the potential role of NF-κB signaling in regulating CD137 expression. Unlike MAPK whose knockdown did not decrease the expression of CD137 in Panc-1 and SW1990 cells, the silencing of NF-κB by siRNA against p65 significantly diminished the CD137 expression in these two cell lines (Fig. 3a, b), suggesting that NF-κB might play a dominant role in regulating CD137 expression in Panc-1 and SW1990 cells. In T-Rex/K-Ras and HCT116 cells, silencing of the NF-κB also led to a significant decrease in CD137 expression (Fig. 3c, d). Altogether, these findings demonstrated the important role of NF-κB in regulating the *CD137* gene transcription, which appears to be a general regulatory mechanism in cancer cells expressing mutant K-Ras.

### The role of IL-1α in mediating K-Ras-induced CD137 expression through NF-κB signaling

**Fig. 4:**
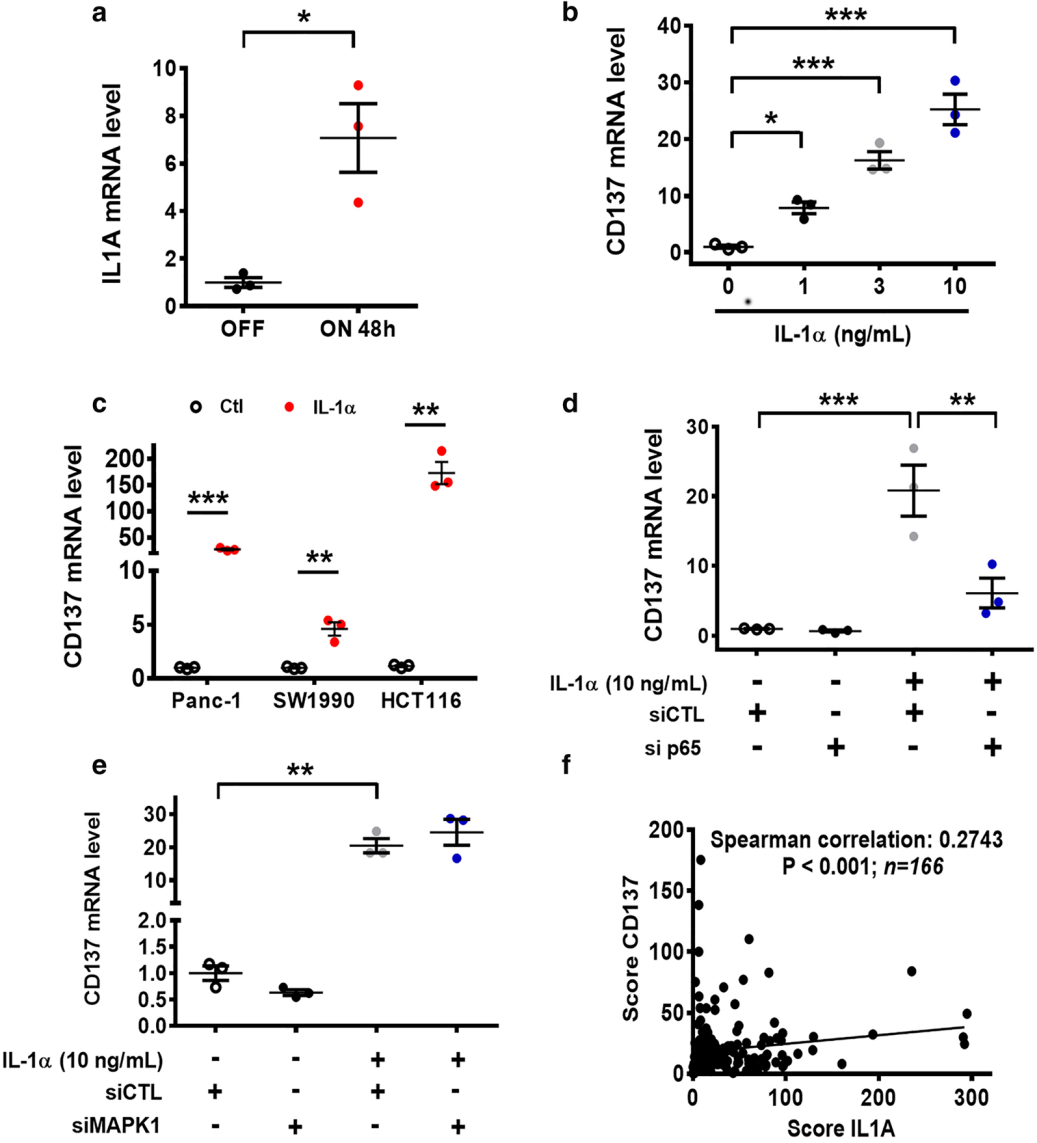
K-Ras-induced CD137 expression is mediated by IL-1α through NF-κB signaling. **a** IL1A mRNA in T-Rex/K-Ras cells measured by real-time PCR. The cells were incubated with 100 ng/mL doxycycline for 48 h. **b** CD137 mRNA expression in T-Rex/K-Ras cells incubated with various concentrations of IL-1α for 24 h. CD137 mRNA was measured by qTR-PCR. **c** CD137 mRNA expression in three human cancer cell lines incubated with 10 ng/mL IL-1α for 24 h. CD137 mRNA was measured by real-time PCR. **d** Impact of NF-κB on IL-1α-induced CD137 expression. T-Rex/K-Ras cells were first transfected with 100 nmol/L control siRNA (siCTL) or siRNA against p65 for 24 h, then incubated with 10 ng/mL IL-1α for 24 h. CD137 mRNA was measured by real-time PCR. **e** Impact of MAPK1 on IL-1α-induced CD137 expression. T-Rex/K-Ras cells were first transfected with 100 nmol/L control siRNA (siCTL) or siRNA against MAPK1 for 24 h, then incubated with 10 ng/mL IL-1α for 24 h. CD137 mRNA was measured by real-time PCR. **f** Relationship between CD137 and IL1A gene expression in 166 human pancreatic tumor samples recorded in the TCGA database. Data are presented as mean ± SEM of three repeated experiments. Statistical analysis: two-tailed unpaired t-test for **a**–**e**. Spearman’s rank correlation test for F. *P < 0.05; **P < 0.01; ***P < 0.001. *CD137* tumor necrosis factor receptor superfamily member 9, *IL1A/IL-1α* interleukin-1 alpha, *siCTL* control siRNA, *siMAPK1* siRNA against MAPK1, *sip65* siRNA against p65, *PCR* polymerase chain reaction

Considering that cytokines play a major role in the activation of the NF-κB pathway and regulation of immune functions, we thus searched for potential cytokine(s) that might mediate K-Ras-induced CD137 expression via the activation of the NF-κB pathway. Real-time PCR analysis revealed a significant increase in IL-1α expression after K-Ras was activated by doxycycline (Fig. 4a). To test the direct impact of IL-1α on the *CD137* gene expression, we incubated T-Rex/K-Ras/off (not incubated with doxycycline) cells with various concentrations of IL-1α for 24 h. CD137 mRNA levels were increased by IL-1α in a dose-dependent manner up to 10 ng/mL, which induced a > 20-fold increase in CD137 mRNA expression in T-Rex/K-Ras/off cells (Fig. 4b). The ability of exogenous IL-1α to stimulate CD137 expression was also observed in Panc-1, SW1990, and HCT116 cells (Fig. 4c). We then investigated if IL-1α mediated CD137 up-regulation through the MAPK signaling or via activating the NF-κB pathway using specific siRNA against the respective pathway components in T-Rex/K-Ras cells. NF-κB silencing significantly abolished the stimulating effect of IL-1α on CD137 expression (Fig. 4d), whereas MAPK1 silencing did not reduce IL-1α-mediated CD137 up-regulation (Fig. 4e). These data together suggest that IL-1α promote CD137 expression mainly through the NF-κB pathway. Indeed, bioinformatics analyses of the TCGA database revealed a positive correlation between CD137 and IL1A gene expression in 166 human pancreatic cancer tissues (*r* = 0.274, *P* < 0.001, Fig. 4f).

**Fig. 5:**
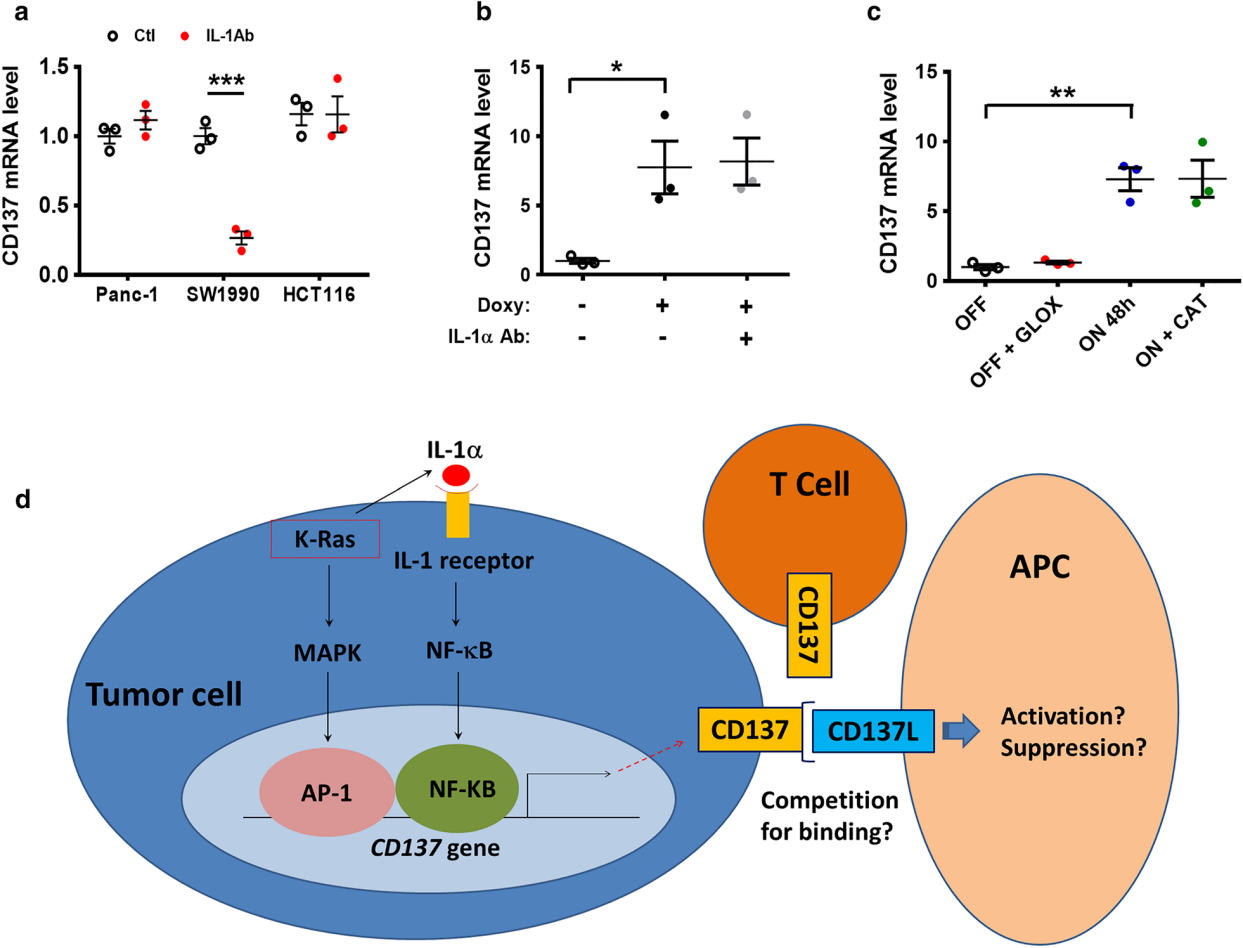
Induction of CD137 expression by K-Ras through MAPK and NF-kB pathways. **a** The indicated three cancer cell lines were incubated with 1 μg/mL neutralizing anti-IL-1α antibody for 48 h. CD137 mRNA was measured by real-time PCR. **b** T-Rex/K-Ras cells were incubated with 100 ng/mL doxycycline and 1 μg/mL neutralizing anti-IL-1α antibody for 48 h. CD137 mRNA was measured by real-time PCR. **c** T-Rex/K-Ras/off cells were incubated with 20 ng/mL glucose oxidase for 48 h. T-Rex/K-Ras/on cells were incubated with 50 μg/mL catalase for 48 h. CD137 mRNA was measured by real-time PCR. **d** Schematic model depicting the likely molecular mechanisms by which K-Ras regulates *CD137* gene transcription. Oncogenic K-Ras signal activates two parallel pathways through MAPK and NF-κB signaling. K-Ras enhances the secretion of IL-1α, which in turn stimulates NF-κB signaling to enhance CD137 expression. Data in **a**–**c** are presented as mean ± SEM of three repeated experiments. Statistical analysis: Two-tailed unpaired t-test for **a**–**c**. *P < 0.05; **P < 0.01. *AP-1* activator protein 1, *APC* antigen-presenting cells, *CAT* catalase, *CD137* tumor necrosis factor receptor superfamily member 9, *CD137L* tumor necrosis factor ligand superfamily member 9, *GLOX* glucose oxidase, *IL-1α* interleukin-1 alpha, *IL-1Ab* neutralizing anti-IL-1α antibody, *K-Ras* V-Ki-ras2 Kirsten rat sarcoma viral oncogene homolog, *MAPK* mitogen-activated protein kinase, *NF-κB* nuclear factor kappa-light-chain-enhancer of activated B cells, *PCR* polymerase chain reaction

We then used a neutralizing antibody against IL-1α to evaluate the role of IL-1α in promoting CD137 expression in various cell lines. Interestingly, this antibody decreased the expression of CD137 only in SW1990 cells (Fig. 5a) and did not significantly impact CD137 expression in Panc-1, HCT116, and T-Rex/K-Ras/on (incubated with doxycycline) cells (Fig. 5a, b). These results suggest that although IL-1α might play an important role in the up-regulation of CD137 via the activation of NF-κB, cancer cells with K-Ras mutations seem to have at least another pathway via MAPK signaling to promote CD137 expression. The relative contribution of each pathway may depend on individual cell lines.

MAPK and NF-kB pathways can be activated by oxidative stress [25, 26], and we have previously demonstrated that K-Ras activation induced ROS (reactive oxygen species) generation in T-Rex/K-Ras inducible cells [21]. To test whether oxidative stress might affect the CD137 expression, we have incubated T-Rex/K-Ras cells (K-Ras off) with glucose oxidase to generate H_2_O_2_ and also incubated the K-Ras/on cells with catalase to reduce H_2_O_2_ in the microenvironment and thus facilitate the flux of H_2_O_2_ out of the cells. As shown in Fig. 5c, no significant change in CD137 expression was observed when the ROS levels were altered, suggesting that oxidative stress did not play a significant role in regulating CD137 expression. Figure 5d illustrates the multifactorial regulation of CD137 in cancer cells driven by K-Ras.

## Discussion

Although the role of CD137 has been intensively studied in immune cells [27], its expression and function in cancer cells are still poorly understood. CD137 expression has been detected in lung tumor [18], leukemia [19], and lymphoma cells [20]. CD137 expression could promote the growth and survival of leukemia [19] and lymphoma cells [20], and inhibit T cell activation. Here, we demonstrated that CD137 could be detected in human pancreatic cancer cells, and oncogenic K-Ras seemed to play a major role in up-regulating CD137. Mechanistically, MAPK and NF-κB pathways mediated the induction of CD137 expression by K-Ras. Although these two signaling pathways have been suggested to regulate CD137 in immune cells [10–12], their involvement in cancer cells has not been previously characterized. Interestingly, previous studies indicated that the CD137 protein level increased when human umbilical vein endothelial cells were treated with IL-1α (10 ng/mL) and tumor necrosis factor alpha (TNF-α) and that CD137 was detected on blood vessel walls [28, 29]. However, no CD137 expression was observed in T cells incubated with IL-1α, suggesting a cell type-dependent mechanism [12].

We observed that for cancer cells with activating K-Ras mutations, the MAPK and NF-κB pathways seem to function in parallel to promote CD137 expression. Although both pathways are driven by K-Ras activation, either pathway is likely to operate independent of the other, as evidenced by a series of specific siRNA knockdown experiments. The NF-κB pathway seems to be mainly activated by IL-1α, whose expression and secretion were enhanced by K-Ras. In contrast, the MAPK pathway is stimulated by K-Ras activation without the involvement of IL-1α. Figure 5c illustrates the working model for the regulation of CD137 expression in the K-Ras-driven cancer cells. It is currently unclear how activation of K-Ras could lead to increased expression of IL-1α. A previous study suggested that IL-1α expression in pancreatic ductal adenocarcinoma could be regulated by p38MAPK [30]. The possibility that K-Ras promotes IL-1α secretion via p38MAPK remains to be investigated.

The relative contributions of MAPK and NF-κB pathways in promoting CD137 are likely cell type-dependent, evident by the findings that specific suppression of each pathway affected CD137 expression differently in different cell types. Both pathways could be active in certain cancer cells, while in some other cells one of the pathways could be dominant. For instance, the expression of CD137 in SW1990 pancreatic cancer cells seems to be mainly regulated by the IL-1α through the NF-κB pathway, since the neutralizing IL-1α antibody could profoundly decrease the expression of CD137 (Fig. 5a). In contrast, the neutralizing IL-1α antibody could not suppress the CD137 expression in Panc-1 cells, indicating that the MAPK pathway might be highly active in Panc-1 cells to promote CD137 expression.

An interesting finding was that IL-1α could stimulate the expression of CD137 in all four cell lines tested (Fig. 4b, c). This would suggest that the neutralizing IL-1α antibody could be a useful tool to modulate CD137 expression, and thus could be employed to impact immune functions in the context of cancer immunotherapy. However, we observed that the neutralization of IL-1α by antibody failed to decrease CD137 expression in 3 out of the 4 cell lines tested (Fig. 5a, b), suggesting that most cancer cells might have multiple pathways to activate the CD137 expression and thus are not highly dependent on IL-1α for stimulation. As such, the design of immunotherapy using IL-1α antibodies should consider this complexity.

The expression of CD137 in immune cells and its normal biological functions have been well characterized, and CD137 agonists have been tested as anticancer agents [27]. However, the cancer-related biological functions of CD137 remain unclear. For instance, the observation that CD137−/− mice exhibited enhanced response to antitumor treatment demonstrated that CD137 signaling in T cells cannot fully explain the clinical benefits of the agonistic CD137 antibodies [31]. In wild-type mice, CD137 expression on regulatory T cells had important role to induce CD137L reverse signaling in antigen-presenting cells (APCs), leading to the generation of M2 macrophages and tumor progression [32]. These contradictory observations make it difficult to predict the impact of CD137 expression in cancer cells on the tumor microenvironment. One possibility would be that CD137 on cancer cell surface functions as a potential competitor of CD137 on T cells by binding with CD137L on the surface of APCs, and thus suppressing immune responses against cancer, as illustrated in Fig. 5d. Conversely, the interaction of CD137 on cancer cell surface with CD137L on APCs might inhibit or modify certain immune responses. Interestingly, a soluble form of CD137 has been detected in the serum of patients with colon cancer [23], leukemia and lymphoma [33]. This secreted form of CD137 could be induced by hypoxia and has been considered as a new mechanism for immune evasion [34]. However, this soluble form was barely detected in the sera of pancreatic cancer patients, and its level was not different from that in the normal sera, suggesting that K-Ras might not be involved in the splicing of CD137 mRNA. As such, the clinical significance of soluble CD137 in pancreatic cancer remains unclear. Obviously, since these questions are highly relevant to cancer immunotherapy, these deserve further study in the future.

## Conclusions

Our study has identified a novel mechanism by which oncogenic K-Ras regulates CD137 in pancreatic cancer cells. The oncogenic signal activates two parallel pathways, namely the MAPK and NF-κB pathways, involving IL-1α stimulation, to enhance the expression of *CD137* in cancer cells.

## Data Availability

The key raw data have been deposited into the Research Data Deposit (http://www.researchdata.org.cn), with the Approval Number of RDDB2019000578 and the datasets used in this study are publicly available.

## Abbreviations

AP-1: activator protein 1
CD137/TNFRSF9: tumor necrosis factor receptor superfamily member 9
CD137L/TNFSF9: tumor necrosis factor ligand superfamily member 9
IL: interleukin
K-Ras: V-Ki-ras2 Kirsten rat sarcoma viral oncogene homolog
MAPK: mitogen-activated protein kinase
NF-κB: nuclear factor kappa-light-chain-enhancer of activated B cells
TNF-α: tumor necrosis factor alpha
